# Medical Society of the County of Erie

**Published:** 1896-02

**Authors:** Franklin C. Gram

**Affiliations:** Secretary


### Society Proceedings.

### MEDICAL SOCIETY OF THE COUNTY OF ERIE.

### *Annual Meeting, January 14, 1896.*

Reported by FRANKLIN C. GRAM, M. D., Secretary.

THE Medical Society of the County of Erie held its annual meeting Tuesday, January 14, 1896, and at the same time celebrated the seventy-fifth anniversary of its existence. The rooms of the Buffalo Academy of Medicine, where the session was held, were inadequate to furnish seating facilities for the large number of physicians who had come to participate in the event.

Dr. J. G. Thompson, of Angola, the vice-president, called the meeting to order at 9.30 A. M., and announced that the president, Dr. Bartlett, was confined to his home by illness and was consequently unable to be present. The minutes of the semi-annual, as well as of the special meeting held in memory of the late Dr. M. B. Folwell, were read and approved.

On motion of Dr. A. A. Hubbell the following resolution was unanimously adopted :

*Resolved,* That the Medical Society of the County of Erie, now in annual session, expresses its sincere sympathy to its president, Dr. F. W. Bartlett, in his distressing illness.

The Vice-president stated that he did not prepare an annual address, as he had expected this to be done by the president, and on motion of Dr. J. J. Walsh he was excused.

Dr. J. B. Coakley, chairman of the Board of Censors, made a verbal report. He stated that Dr. King, whose case was referred to at the June meeting, had suddenly disappeared as a sequel to the censors' efforts. They had found no more irregularities in Marilla, and had received word that a physician was illegally practising medicine in North Evans, but on investigation found that this was not the case. Complaint was also made against Dr. Weinbach, of this city, who was said to have no diploma. On investigation the board found that he had actually three diplomas and a license to practise. A case from Tonawanda was also referred to the board.

This man afterward located in Buffalo and said he did not use materials, but inherited from his father power to control disease. He claimed not to practise in violation of law, but the District Attorney, before whom the question was laid, thought otherwise, and the case is now before the Grand Jury. A Dr. Jones had also left town through the board's efforts. Another case of a clairvoyant doctress was still under advisement. All the expenses incurred were those of securing a copy of the list of qualified practitioners who were registered with the County Clerk, and this amounted to $2.00. He commended the District Attorney on his willingness to assist the board at all times.

The report was received and the thanks of the society extended to the board for its efforts.

Dr. LUCIEN HOWE bore testimony to the willingness of the District Attorney to coöperate in the prosecution of violations of the medical law. A midwife had just been convicted for failing to report a case of ophthalmia neonatorum—but as it was the first conviction under this law the justice suspended sentence.

Dr. LUCIEN HOWE, chairman of the special committee on arrangements for the seventy-fifth anniversary, made a report of what the committee had done, as shown by the program and circulars sent the members.

Dr. G. W. MCPHERSON, chairman of the committee on membership, reported in favor of admitting the following: Drs. R. H. Lounsbury, John V. Woodruff, F. H. Milliner, Amelia F. Dresser, Jacob Miller, C. G. Fisher, Ray H. Johnson and John E. Bacon. The report was adopted and the new members were introduced to the society.

Applications were received from the following, who desired to become members: Drs. Martha F. Caul, Wellington G. Grove, Richard H. Satterlee, J. Grafton Jones, Simon Clug, John R. McCartey, Chester T. Stewart, E. E. Blaauw, F. E. Luke, Henry Osthues, H. C. Rooth, J. Henry Dowd and J. J. Finnerty. Referred to the committee on membership.

Dr. EDWARD CLARK, the treasurer, made his annual report, which showed the following:

Cash on hand December 31, 1894, $287.58; received for dues in 1895, $217; total, $504.58.

The expenditures were $221.95, leaving a balance in the treasury, December 31, 1895, of $282.63.

The report was received and referred to an auditing committee, consisting of Drs. J. J. Walsh and Wm. W. Potter, who reported having found the accounts and vouchers correct, and recommended that the usual sum of $50 each be allowed the secretary and treasurer for their services during the past year. The recommendation was adopted.

A committee on nominations, consisting of Drs. J. B. Coakley, Geo. Abbott, Henry Lapp, Wm. C. Krauss and J. C. Greene, was appointed. They reported in favor of the following, who were thereupon duly elected:

President, Dr. J. G. Thompson, of Angola; vice-president, Dr. Henry R. Hopkins, Buffalo; secretary, Dr. Franklin C. Gram, Buffalo; treasurer, Dr. Edward Clark, Buffalo; censors, Drs. J. B. Coakley, M. Hartwig, Benjamin G. Long, F. S. Metcalfe, Buffalo, and Henry Lapp, Clarence.

Committee on Membership—Drs. G. W. McPherson, T. M. Johnson and H. E. Hayd.

This committee also reported in favor of appointing a special committee, with Dr. Lucien Howe as chairman, to coöperate with similar committees from other bodies, for the purpose of making arrangements for the reception and entertainment of the American Public Health Association, which will meet in Buffalo in September, 1896. This recommendation was adopted and the chair appointed Drs. Lucien Howe, P. W. Van Peyma, Ernest Wende, W. H. Gail and C. C. Frederick.

Dr. J. B. Coakley moved that Dr. W. W. Potter be appointed a committee of one to confer with the District Attorney with reference to the medical law, which is at present somewhat ambiguous in its construction, the object being to consider the advisability of so amending it to make its meaning clear. The motion was carried.

Dr. William Warren Potter offered the following:

*Resolved*, That Drs. Ernest Wende, Herman Mynter, J. G. Thompson, Henry Lapp and Walter D. Greene be and are hereby appointed a committee to consider the propriety and investigate the feasibility of establishing a professional home for the several medical societies of the County of Erie and the City of Buffalo, this committee to have full power to secure pledges of money or contributions for such a purpose, and to report the result of its labors at the semi-annual meeting in June, 1896.

In support of the resolution, Dr. Potter said that such a representative body as the physicians of Erie County ought to have a permanent home, which should be in keeping with the standing of the profession.

He said, further, that he had called attention to this subject in his presidential address three years ago; that though no action yet had been taken, it was by no means a dead letter; that smaller cities than Buffalo enjoyed the benefits of such comforts and that the time had fully arrived, in his judgment, for the medical profession of Erie County to take action in reference to the establishment of a suitable home for itself. The committee, he said, is a representative one and would no doubt decide wisely as to what had better be done. He moved the adoption of the resolution.

The motion was seconded by Dr. Coakley and unanimously adopted.

The Secretary stated that he had received a communication from Dr. F. C. Curtis, secretary of the medical society of the State of New York, stating that there is some doubt about the incorporation of this society and its ability to maintain a legal action. A law had been passed in 1894—a copy of which was submitted—, to remedy similar cases, and he advised the society to take advantage of that law. On motion of Dr. F. S. Crego the entire matter was referred to the president and secretary, with power.

Dr. M. D. Mann read a resolution adopted by the Lake Erie Medical Society, requesting the State Board of Health to appoint a competent physician to investigate the causes and continuation of tuberculosis among the Indians of New York State, especially among those residing on the Cattaraugus Reservation in Erie County. He spoke in favor of indorsing this action, which was done, and the president and secretary were instructed to sign the resolution and forward it to the State Board of Health at Albany.

Dr. Geo. F. Cott read a paper by title on Some unavoidable accidents of intubation. He exhibited the necessary instruments and explained the mode of using them.

Dr. F. C. Gram then read a paper giving a synopsis of the history of the society for the past seventy-five years. On motion of Dr. Edward Storck he was given a vote of thanks. Dr. J. W. Putnam considered that a paper of such historical importance should be preserved and also distributed among the members. He moved that it be published in the Buffalo Medical Journal, and that the society order five hundred reprints in pamphlet form for distribution. This motion was adopted, and later the society added to this all papers of an historical nature read at this anniversary meeting.

Dr. John Hauenstein was received with applause when he stepped on the platform to read a paper on The first uses of chloroform and ether in Buffalo. This was repeated when Dr. Wyckoff presented his paper on The establishment and early days of the Medical Department of the University of Buffalo, and when Dr. John Cronyn read a paper on The establishment of the Medical Department of Niagara University. Dr. Samo's paper on Cholera epidemics in Buffalo was not presented, as the author desired further time in which to complete it. All these papers will be published in full.

Dr. Edward Storck offered the following resolution, and moved its adoption :

*Whereas*, Medical men are especially interested in the proper management of hospitals, the profession of this county, represented in the Erie County Medical Society, recommends and urges the removal of the Erie County Hospital from political influences, its divorce from the almshouse and its economical and efficient administration by a management qualified for the task.

The resolution brought out a warm discussion, which was participated in by Drs. Porter, Walsh, Pryor, J. C. Greene, Howell, Cronyn, Wyckoff and Storck.

An amendment by Dr. Porter to table the resolution was lost, whereupon Dr. Walsh moved to refer the matter to a special committee of five, to report at the June meeting. Dr. Walsh's amendment was carried. The president stated that he would appoint the committee later.

Shortly after 1 o'clock P. M. the society adjourned.

